# Effectiveness of the interventions against workplace violence suffered by health and support professionals: A meta-analysis

**DOI:** 10.1590/1518-8345.5923.3639

**Published:** 2022-08-12

**Authors:** Caroline Vieira Cláudio Okubo, Júlia Trevisan Martins, Tatiana da Silva Melo Malaquias, Maria José Quina Galdino, Maria do Carmo Fernandez Lourenço Haddad, Alexandrina Aparecida Maciel Cardelli, Renata Cristina de Campos Pereira Silveira

**Affiliations:** 1 Universidade Federal do Paraná, Complexo Hospital de Clínicas, Unidade Cirúrgica, Curitiba, PR, Brasil.; 2 Universidade Estadual de Londrina, Departamento de Enfermagem, Londrina, PR, Brasil.; 3 Universidade Estadual do Centro-Oeste, Departamento de Enfermagem, Guarapuava, PR, Brasil.; 4 Universidade Estadual do Norte do Paraná, Departamento de Enfermagem, Bandeirantes, PR, Brasil.; 5 Bolsista do Conselho Nacional de Desenvolvimento Científico e Tecnológico (CNPq), Brasil.; 6 Universidade de São Paulo, Escola de Enfermagem de Ribeirão Preto, Centro Colaborador da OPAS/OMS para o Desenvolvimento da Pesquisa em Enfermagem, Ribeirão Preto, SP, Brasil.

**Keywords:** Evidence-Based Practice, Systematic Review, Meta-Analysis, Occupational Exposure, Workplace Violence, Health Personnel, Prática Clínica Baseada em Evidências, Revisão Sistemática, Metanálise, Exposição Ocupacional, Violência no Trabalho, Pessoal de Saúde, Práctica Clínica Basada en la Evidencia, Revisión Sistemática, Metaanálisis, Exposición Profesional, Violencia Laboral, Personal de Salud

## Abstract

**Objective:**

to assess the effectiveness of the interventions targeted at preventing and reducing the workplace violence suffered by health and support professionals.

**Method:**

a systematic review with meta-analysis conducted in eight databases and in the gray literature. Risk of bias was assessed by means of the Cochrane tools and certainty of the evidence, through *Grading of Recommendations Assessment, Development and Evaluation*. The analysis was performed in a descriptive manner and through the meta-analysis, including a heterogeneity assessment.

**Results:**

a total of 11 randomized and quasi-randomized studies were eligible, of which six (54.5%) implemented individual skills, four used a multiple approach (36.4%) and one (9.1%) resorted to governmental actions. Four studies (36.4%) exerted a positive and significant effect on reducing violence. Risk of bias was classified as high or uncertain. The meta-analysis was performed with two studies that tested individual skill (intervention group) *versus* individual skill (comparator group), although there was no scientific evidence (95% CI: -0.41 - 0.25, p=0.64) for the violence prevention/reduction outcome.

**Conclusion:**

this review did not obtain a high level of evidence in the prevention or reduction of workplace violence. The reduced number of randomized trials, the lack of studies with low risk of bias and the high consistency may have been factors that hindered recommending effective interventions.

## Resumo

**Objetivo:**

avaliar a efetividade das intervenções direcionadas à prevenção e redução da violência no trabalho sofrida por profissionais de saúde e apoio.

**Método:**

revisão sistemática com metanálise realizada em oito bases de dados e na literatura cinzenta. O risco de viés foi realizado por meio das ferramentas da Cochrane e a certeza da evidência pelo *Grading of Recommendations Assessment, Development and Evaluation*. A análise foi realizada de forma descritiva e pela metanálise, incluindo avaliação da heterogeneidade.

**Resultados:**

11 estudos aleatorizados e quasi-aleatorizados foram elegíveis, dos quais seis estudos (54,5%) implementaram habilidades individuais, quatro multiabordagem (36,4%) e um (9,1%) ações governamentais. Quatro estudos (36,4%) tiveram efeito positivo e significativo na redução da violência. O risco de viés foi classificado como alto ou incerto. A metanálise foi realizada com dois estudos que testaram habilidade individual (grupo intervenção) *versus* habilidade individual (grupo comparador), porém não houve evidência científica (IC 95%: -0,41 a 0,25, p=0,64) para o desfecho prevenção/redução da violência.

**Conclusão:**

esta revisão não obteve alta evidência na prevenção ou redução da violência no trabalho. O número reduzido de ensaios aleatorizados, a falta de estudos com baixo risco de viés e a alta consistência podem ter sido fatores dificultadores para recomendar intervenções efetivas.

## Resumen

**Objetivo:**

evaluar la efectividad de las intervenciones dirigidas a prevenir y reducir la violencia laboral que sufren los profesionales de la salud y de apoyo.

**Método:**

revisión sistemática con metanálisis realizada en ocho bases de datos y en la literatura gris. El riesgo de sesgo se evaluó mediante herramientas Cochrane y la certeza de la evidencia mediante el *Grading of Recommendations Assessment, Development and Evaluation*. El análisis se realizó de forma descriptiva y por metanálisis, e incluyó la evaluación de la heterogeneidad.

**Resultados:**

fueron elegibles 11 estudios aleatorios y cuasialeatorios, de los cuales seis estudios (54,5%) implementaron habilidades individuales, cuatro multienfoque (36,4%) y uno (9,1%) acciones gubernamentales. Cuatro estudios (36,4%) tuvieron un efecto positivo y significativo en la reducción de la violencia. El riesgo de sesgo se clasificó como alto o incierto. El metanálisis se realizó con dos estudios que evaluaron la capacidad individual (grupo de intervención) *versus* la capacidad individual (grupo de comparación), pero no se encontró evidencia científica (IC del 95 %: -0,41 a 0,25, p = 0,64) para el resultado prevención/reducción de la violencia.

**Conclusión:**

esta revisión no obtuvo alta evidencia sobre la prevención o reducción de la violencia laboral. El número reducido de ensayos aleatorios, la falta de estudios con bajo riesgo de sesgo y la alta consistencia pueden haber sido factores que dificultaron la recomendación de intervenciones efectivas.

**Highlights**

(1) The paper synthesizes the knowledge about the interventions that prevent workplace violence.

(2) The interventions implemented in the studies can benefit health professionals.

(3) The paper raises awareness regarding the theme in health professionals and managers.

(4) It is recommended to conduct new and in-depth randomized clinical trials.

## Introduction

Workplace violence is significantly and increasingly present in the world. In the United States of America, a report published in 2017 by the United States Bureau of Labor Statistics revealed that 458 homicides were recorded and committed in the workplace, of which 77 were perpetrated by co-workers or associates^1^.

In 2018, data revealed that health care professionals were five times more likely to experience workplace violence than all other workers, accounting for nearly 73% of all non-fatal work-related accidents and diseases requiring days off^2^.

A systematic review conducted in 2019 revealed that 80% of all the cases of workplace violence affected health professionals from Asia, America, Europe, Middle East, Oceania and Africa^3^. In 2021, a report published by the Joint Commission on sentinel events from 2018 to 2020 revealed that, in relation to the rape of health professionals in the United States of America, 56 events were recorded, in addition to 12 homicides and 260 suicides^4^.

In this study, it is understood that workplace violence consists of intentional acts or aggressive and threatening behaviors that deviate from the expected actions and seek to harm or injure a person during work or as a result of it, including verbal, non-verbal, threatening or demeaning words or actions, bullying, sexual harassment, physical assaults or other intimidating or disruptive behavior involving professionals, patients or visitors^2^ ^,^ ^5^.

A systematic review with meta-analysis verified that nearly 2% of the health professionals are victims of workplace violence. Regarding the type of violence, predominance of verbal abuse was evidenced, followed by verbal threats and sexual harassment. In relation to the practice locus, there was higher prevalence in pre-hospital environments^6^.

The aforementioned type of violence has been indicated as a priority area from 2002 to the present day, and political intervention at the international level is a concern, mainly in the health sector, because, among all, it is one of the most affected, exerting negative effects on work productivity, quality of the care provided to the patient and the costs, in addition to the high rates of absenteeism and abandonment of the profession^2^ ^,^ ^7^. 

A number of studies also reveal that the aforementioned violence in the health sector especially affects female professionals and the Nursing category that practices their profession, especially in hospitals, emergency departments and without another co-worker^8^ ^-^ ^11^.

In order to combat this complex phenomenon, several institutions and international bodies have been publishing guidelines to eliminate it with a focus on a zero tolerance culture, addressing measures to minimize or exclude the workplace violence rates and risk. Such measures include commitment by the management, participation of the professional, workplace analysis, safety and health training, risk factor analysis and monitoring records of the violence rates^2^ ^,^ ^7^ ^-^ ^8^.

Thus, actions and implementation of guidelines, laws or public policies to reduce violence should be sought, as it is a preventable problem and an important determinant of physical illness and, above all, mental ailments^12^. 

Among the systematic reviews that sought to identify the effect of the interventions, it was verified that there is lack of knowledge in the studies about the effectiveness of the actions that prevent or reduce acts of violence against professionals working in the health services. It should be noted that one study only evaluated a specific intervention at the individual level, including education and training^13^ and another did so at the organizational level, such as work programs and practices^14^.

In view of the aforementioned considerations, this study is justified, as identifying the scientific evidence on the theme will contribute to the standardization of effective interventions that may curb and prevent acts of violence that affect health and support professionals.

Thus, the objective of this systematic review was to assess the effectiveness of the interventions targeted at preventing and reducing the workplace violence suffered by health and support professionals.

## Method

### Study design

This study is a systematic review with meta-analysis written according to the Preferred Reporting Items for Systematic Reviews and Meta-Analyses (PRISMA)^15^ ^-^ ^17^. The protocol of this review was registered in the International Prospective Register of Systematic Reviews (PROSPERO) platform under number CRD42018111383^18^. This protocols was also published in the BMJ Open Journal^19^.

### Selection criteria

To search for studies and formulate the guiding question, the PICOS^20^ strategy was used, an acronym for *“Population”* (health and support professionals), *“Intervention”* (organizational, environmental, individual, multiple approach [organizational, environmental and individual] or governmental [policies/laws]), *“Control/Comparison”* (no comparison, standard, usual or no intervention is applicable/eligible) and *“Outcome”* (prevention and reduction of workplace violence or reduction of exposure to this type of violence).

This review had the following guiding question: How effective are the interventions targeted at preventing and reducing the workplace violence suffered by health and support professionals? 

The inclusion criteria adopted addressed studies: 1) conducted with health and/or support professionals^21^; 2) carried out in health services or community health services, such as hospitals, emergency sectors, basic health units or long-term care institutions, in addition to the patient’s home; 3) addressing organizational, environmental, individual, multiple approach (organizational, environmental and individual) or governmental interventions^7^; 4) that had reduction and/or prevention of workplace violence perpetrated by patients as primary or secondary outcomes; 5) with randomized or quasi-randomized designs such as randomized clinical trials (level II evidence) and quasi-randomized studies (such as before-and-after type with a control group, level III evidence)^22^ ^-^ ^24^. No restriction was applied in relation to language or year publication.

The exclusion criteria adopted addressed studies: 1) conducted with residents and/or students; 2) with review methodologies, letters, personal opinions, book chapters, institutional manuals, reports, case series, cross-sectional studies (non-comparative as the before-and-after type without control group); and 3) with duplicate data.

### Period

Data collection took place during 2020 and 2021. A search in the databases was conducted on August 8^th^, 2020. Another updated search was performed on June 9^th^, 2021.

### Data collection

The individual search strategies for each electronic database were implemented in PubMed, Scopus, Web of Science, EMBASE, the Cochrane Library, CINAHL, LILACS and Livivo. In addition to that, searches were also conducted in the gray literature, including Google Scholar, OpenGrey and ProQuest. The strategy was prepared by the research team of this review, including PhDs on the theme and on the review method.

It is noted that, prior to carrying out the final searches of the primary studies in the databases selected, several combinations were made employing the controlled descriptors, keywords and the AND and OR Boolean operators. The objective was to identify the highest possible number of publications, first in the PubMed database with adaptation for the others. The *MeSH* descriptors included the following: “health personnel”, “attitude of health personnel”, “workplace violence”, “exposure to violence”, “physical abuse” and “education”, in addition to synonyms and keywords. The search was conducted by two researchers with PhD degrees on the theme of violence, as well as on the method adopted for the study. 

A manual search was also conducted in the references of all the articles included. An expert on the topic of “workplace violence” was identified via a website (http://expertscape.com/), contacted by email and asked to identify the five most important publications on the topic.

The studies were first exported by a PhD to EndNote online^25^, where a detailed screening of all studies and references was performed and duplicates were removed. Subsequently, the citations were exported by the same PhD to the Rayyan QCRI manager^26^, with a new process for removal of duplicates; and selection of the studies, in two phases, was performed by two reviewers.

In the first selection phase, two masked reviewers (one with a Master’s degree and the other with a PhD) independently read and evaluated the titles and abstracts of all studies, applying the eligibility criteria to define the studies to be included. In the second phase, these same reviewers read the full texts to confirm eligibility. 

Data extraction and collection was in charge of another two reviewers (with Master’s and PhD degrees, respectively), masked and by means of a form. This form contained a number of study characteristics (author, year, country, study design, objective, locus, study period), population (category, gender, sample size), characteristics of the results (intervention and control groups, including the total sample number of these groups (n) and a description of the intervention and control, randomization, blinding, main results) and main conclusion. A maximum of three attempts were made to contact the authors of the studies to retrieve the information. Subsequently, data accuracy was confirmed between the reviewers.

Any and all disagreements were resolved in a meeting between both reviewers. If no consensus was reached, another two reviewers (PhDs) with expertise in the topic of worker’s health and in the method were contacted to resolve the differences in the aforementioned phases (data collection, selection of the studies and/or extraction).

### Data treatment and analysis

Risk of bias of the studies selected was assessed by means of the following Cochrane tools: Revised Cochrane Risk-of-Bias Tool for Randomized Trials (RoB 2), ROB 2 for Cluster - Randomized Trials (RoB 2 CRT) and Risk Of Bias In Non-randomised Studies - of Interventions (ROBINS-I)^27^ ^-^ ^30^.

Two masked reviewers (master’s degree and PhD) evaluated each domain (selection, performance, detection, attrition and reporting) and classified each study as high risk, low risk or some concerns regarding bias, based on the aforementioned tools. 

A synthesis of the results obtained was performed in a descriptive manner and through the meta-analysis. The results of the meta-analysis were presented in a forest plot. The evaluations of the measures of the continuous outcomes adopted provided a general estimate, through the standardized mean difference, weight for the model adopted (random), and with a 95% Confidence Interval (CI). The meta-analysis was performed by means of the Cochrane’s Review Manager (V.5.3) - RevMan Web software^31^ ^-^ ^32^. Heterogeneity was also described, by means of the I^2^statistical test^31^. No sensitivity analysis was applied due to the limitation of two randomized clinical trials in the meta-analysis.

A summary of the overall certainty of the evidence for the outcome studied (prevention/reduction of workplace violence) was assessed by two masked reviewers (master’s degree and PhD), who grounded their evaluations on the Grading of Recommendations Assessment, Development and Evaluation approach, GRADE)^33^ and on the GRADEpro GTD software (Copenhagen, Denmark) provided by the GRADE Working Group, in association with the Cochrane Collaboration^34^.

As in the other stages, other reviewers (two PhDs with expertise in systematic reviews and in the review method) were consulted for the disagreements about data treatment and analysis.

## Results

In the first phase of this review, 4,909 citations were identified in eight databases, mentioned in the method. Subsequently, after removing the 1,963 duplicate citations, the titles and abstracts of 2,946 articles were assessed to apply the eligibility criteria. Thus, 2,903 studies were included in phase 1. Complementarily, searches were conducted in the gray literature, the reference lists of the articles included were read and the experts were consulted, with addition of another 144 articles to the first phase. Of these, nine were included for the second phase: full-reading.

**Figure 1:**
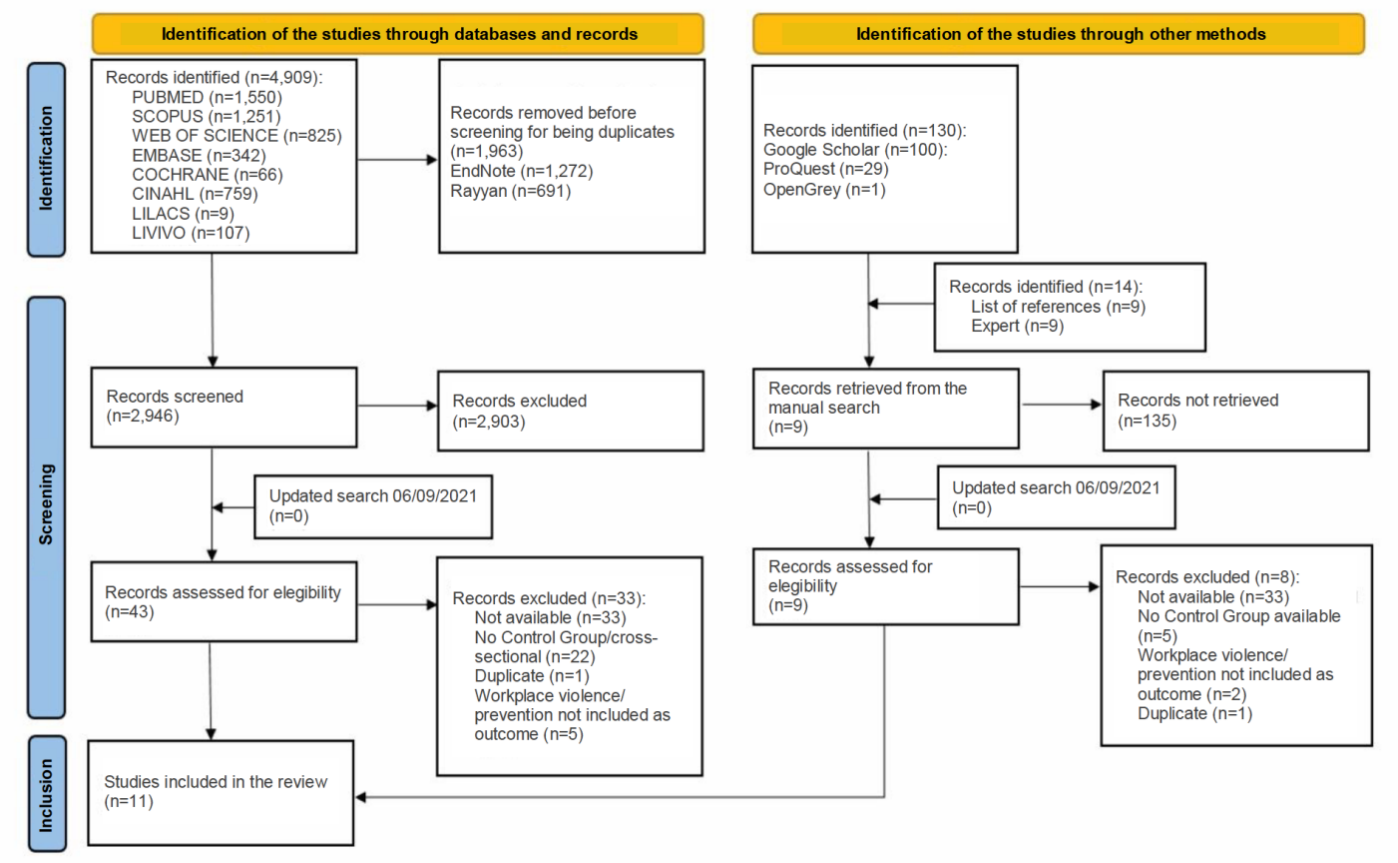
Flowchart of the process corresponding to the identification, inclusion and exclusion of the studies, according to the Preferred Reporting Items for Systematic Reviews and Meta-Analyses. Londrina, PR, Brazil, 2021

Source: Adapted from Page, et al.^17^.

A total of 52 articles (43 from the databases and 9 found through other methods) were eligible for the second phase. Of these, 41 were excluded after full-reading. An updated search, carried out on 06/09/2021, provided two studies to be screened for full-text reading, but they were excluded after adoption of the eligibility criteria. Therefore, 11 studies were included for the descriptive analysis. Figure 1 presents a detailed flowchart of the process corresponding to the identification, inclusion and exclusion of studies according to the PRISMA guidelines^17^.

The main descriptive characteristics of the articles included are presented in Figure 2.

**Figure 2:** Studies included in the systematic review according to authors, year of publication, method, sample (n), Intervention Group (n), Control Group (n) and main conclusion (n=11). Londrina, PR, Brazil, 2021

Authors/Year	Method	Sample (n)	IG*(n)	CG^†^ (n)	Main conclusion
Arnetz, Arnetz^35^ 2000	Quasi-randomized	EDs^‡^, geriatric, psychiatric and home care (n=47)	Individual skills (structured program, feedback groups) (n=24)	None (n=23)	The program did not reveal any statistically significant difference in self-reported WPV^§^.
Gates, Fitzwater, Succop^36^ 2005	Quasi-randomized	Professionals (n=138)	Individual skills (lectures, discussions with videos, demonstrations and problem-solving) (n= 53)	None (n=49)	The intervention did not exert any significant effect on the incidence of aggressions.
Anderson^37^ 2006	Quasi-randomized	Professionals (n=43)	Individual skills (online training containing risk assessment, assertiveness techniques, and ethical and legal questions) (n=22)	None (n=21)	Only verbal abuse was statistically significant between the IG* (intervention concluded in 30 days) and the CG^†^, with a decline in the number of events.
Casteel, et al.^38^ 2009	Quasi-randomized	EDs^‡^ (n=166)	Governmental (California State Law for Safety Protection) (n=116)	Governmental (only OSHA^||^ guidelines) (n=50)	The policy (state law) can be an effective method to improve health professionals’ safety.
Kling, et al.^39^ 2011	Quasi-randomized	High-risk patients (n=473)	Multiple approach (Training of an electronic patient alert system, containing risk assessment and courses of action after signaling those at risk of WPV^§^, such as nearby security guards) (n=109)	None (n=634)	The Alert System did not prevent WPV^§^ incidents by the patients after being signaled, as the rates only decreased during the implementation period for this system.
Irvine, et al.^40^ 2012	Cluster *RCT* ^¶^	Long-term care institution (n=6)	Individual skills (immediate training containing programs and courses through videos and demonstrations) (n=3)	Individual skills (delayed training containing programs and courses through videos and demonstrations) (n=3)	Training through the Internet was an effective tool to reduce the WPV^§^ rates, and the effects of training can improve with time.
Gillespie, et al.^41^ 2014	Quasi-randomized	EDs^‡^ (n=6)	Multiple approach (meetings with feedback and environmental and organizational changes) (n=3)	None (n=3)	Two IG* loci presented a significant reduction in WPV^§^.
Glass, et al.^42^ 2017	RCT^¶^	Professionals (n=306)	Individual skills (computer-based training without peer facilitation - definition and prevalence of WPV^§^, assertiveness techniques) (n=154)	Individual skills (computer-based training with peer facilitation) (n=52)	Computer-based training with or without peer facilitation was able to reduce the number of WPV^§^ incidents.
Arnetz, et al.^43^ 2017	Cluster *RCT* ^¶^	EDs‡^‡^ and psychiatrics, among others (n=36)	Multiple approach (participant action-research with individual, environmental and organizational aspects) (n=19)	None (n=17)	The data-based intervention was effective and significant in reducing the WPV^§^ risks and related injuries.
Sadatmahaleh, et al.^44^ 2018	Quasi-randomized	Professionals (n=48)	Multiple approach (WPV^§^ management program through workshops, group discussions and lectures) (n=24)	None (n=24)	WPV^§^ frequency was reduced in the IG*, although the reduction was not statistically significant between the groups.
Baby, Gale, Swain^45^ 2019	Cluster *RCT* ^¶^	Professionals (n=127)	Individual skills (group training for communication skills, realistic situations about WPV^§^) (n=64)	Individual skills (group training addressing mindfulness techniques) (n=63)	The effect between the IG* and the CG^†^ did not present any statistically significant difference.

*IG = Intervention Group; ^†^CG = Control Group; ^‡^EDs = Emergency Departments; ^§^WPV = Workplace Violence; ^||^OSHA = Occupational Safety and Health Administration; ^¶^RCT = Randomized Clinical Trial.

The studies included were conducted in five countries: Sweden^35^, United States of America^36^ ^-^ ^38^ ^,^ ^40^ ^-^ ^43^, Canada^39^, Iran^44^ and New Zealand^45^, with most of them (63.7%) from the United States of America. The studies were published between 2000 and 2019.

Regarding the methodology adopted, seven (63.7%) studies^35^ ^-^ ^39^ ^,^ ^41^ ^,^ ^44^ were classified as quasi-randomized and four (36.3%) studies^40^ ^,^ ^42^ ^-^ ^43^ ^,^ ^45^ as randomized clinical trials, one of the classic type^42^ and three of the cluster type^40^ ^,^ ^43^ ^,^ ^45^. Duration of these studies did not present a pattern, but varied between four and 1,440 months, and the intervention took place between one and 360 months after the beginning of the study.

The studies were carried out in different health services, with predominance of hospitals (54.5%)^37^ ^-^ ^39^ ^,^ ^41^ ^,^ ^43^ ^-^ ^44^, followed by hospital emergency services^35^ ^,^ ^38^ ^,^ ^41^ ^,^ ^44^, psychiatric services^35^ ^,^ ^38^, home units^35^ ^,^ ^42^, long-term care or geriatric institutions^35^ ^-^ ^36^ ^,^ ^40^, non-governmental organizations and District Health Councils^45^.

As for the categories of professionals in the studies included, most of them (n=9; 81.9%) were developed with the Nursing team^35^ ^-^ ^37^ ^,^ ^39^ ^-^ ^44^, including nurses^35^ ^,^ ^37^ ^,^ ^41^ ^,^ ^43^ ^-^ ^44^, nursing technicians^35^ ^-^ ^37^ ^,^ ^41^ ^,^ ^43^, nursing assistants^37^ ^,^ ^40^ ^-^ ^41^ ^,^ ^43^ and homecare - nursing assistants^42^. The studies were also carried out with physicians^41^, paramedics^41^, health managers^43^, health support workers - security, secretaries^39^ ^,^ ^43^ ^,^ ^45^, rehabilitation/physiotherapy workers, laboratories and radiology^37^, in addition to health workers that were not specified^38^.

The total number (n) of professionals described above was 4,790. There was predominance of the female gender in eight studies (72.8%), although three (27.2%) surveys^35^ ^,^ ^38^ ^-^ ^39^ did not report the prevailing gender. Some studies reported the total mean age and others did so by group or predominant age interval, thus hindering presentation of these results.

The study sample for composition of the Intervention Group (IG) and Control Group (CG) varied across professionals, departments and patients, with five (45.5%) studies^36^ ^-^ ^37^ ^,^ ^42^ ^,^ ^44^ ^-^ ^45^ having the professionals as sample, another five (45.5%)^35^ ^,^ ^38^ ^,^ ^40^ ^-^ ^41^ ^,^ ^43^, the departments and only one (9%)^39^, the patients.

The IG interventions covered three criteria: individual skills development, multiple approach (organizational, environmental and individual) and governmental actions (law/policy implementation), while the CG covered individual skills, governmental action and no intervention. Both classifications were defined based on the International Labor Organization recommendations^7^.

In the IG, six studies (54.5%) implemented interventions for the development of individual skills^35^ ^-^ ^37^ ^,^ ^40^ ^,^ ^42^ ^,^ ^45^, four (36.4%) resorted to the multiple approach^39^ ^,^ ^41^ ^,^ ^43^ ^-^ ^44^ and only one (9.1%) to governmental actions (state law)^38^.

For the CG, the majority, seven studies (63.6%), did not implement any intervention^35^ ^-^ ^37^ ^,^ ^39^ ^,^ ^41^ ^,^ ^43^ ^-^ ^44^, three (27.3%) developed individual skills as a control^40^ ^,^ ^42^ ^,^ ^45^ and only one (9.1%) implemented governmental intervention (federal policy/guideline)^38^. Of them, four (36.4%) exerted a positive and significant effect on reducing violence^37^ ^-^ ^38^ ^,^ ^40^ ^,^ ^43^.

**Figure 3:**
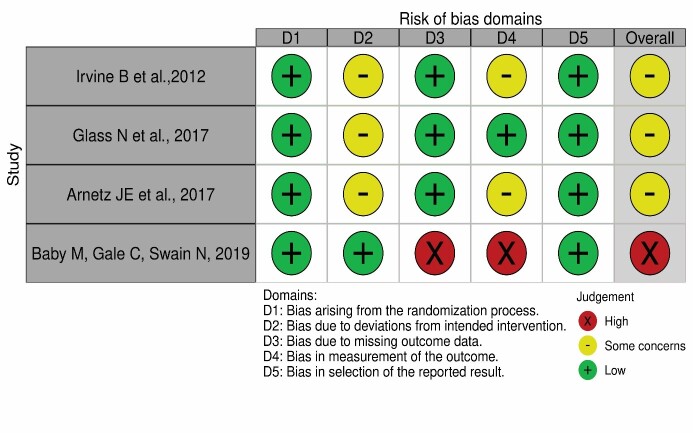
Risk of bias assessment corresponding to the randomized clinical trials in each domain of the Revised Cochrane Risk-of-Bias Tool for Randomized Trials and for Cluster - Randomized Trials tools Londrina, PR, Brazil,2021

Source: Figure generated in the Robvis^®^ tool^46^ application. Prepared by the author (2021)

Figure 3 presents the result of the risk of bias assessment corresponding to the randomized clinical trials through the RoB 2 and RoB 2 CRT tools^27^ ^-^ ^28^.

In relation to the randomized clinical trials, of the four studies included, three (75%) were classified as (un)certain risk^40^ ^,^ ^42^ ^-^ ^43^, while one (25%) was categorized as high risk^45^ due to concerns about deviations from the intended intervention (domain 2), missing data (domain 3) and measurement of outcomes (domain 4).

Regarding the seven quasi-randomized studies analyzed by means of ROBINS-I^29^ ^-^ ^30^, five (71.4%) were classified as serious risk^34^ ^,^ ^37^ ^,^ ^39^ ^,^ ^41^ ^,^ ^44^, one (14.3%) as moderate risk^38^ and another one (14.3%) as uncertain risk^36^. These studies presented significant biases in domain 1 (confounding factors), domain 3 (classification of the intervention) and domain 6 (measurement of the outcomes).

In order to assess the possibility of meta-analysis, the first step was to group the studies according to the PICOS acronym. Thus, the (methodologically and clinically) homogeneous studies were combined, resulting in the meta-analysis of two studies^40^ ^,^ ^42^. Heterogeneity was considered important (I^2^=88%). The estimate corresponding to the mean of the random effect of the studies was -0.08 and the meta-analysis diamond 95% CI varied from -0.41 to 0.25, with p=0.64.

**Figure 4:**
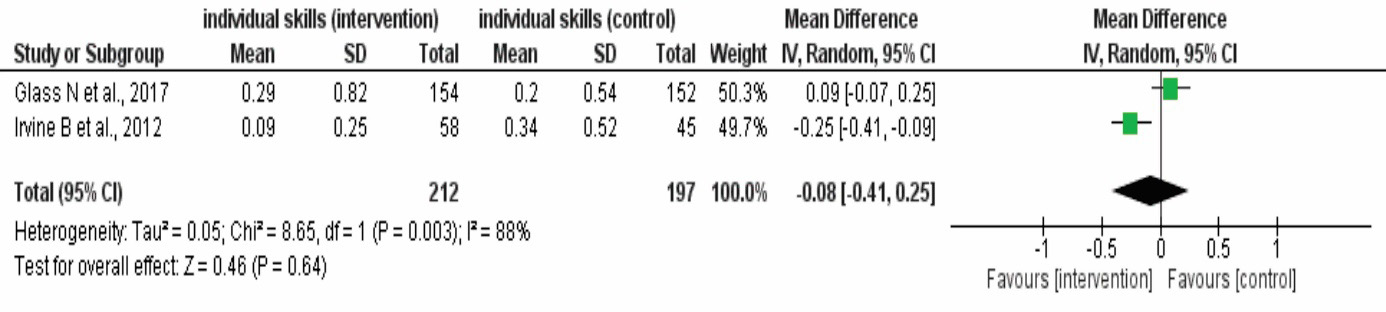
Forest plot corresponding to the meta-analysis of the individual skills (intervention) *versus* the individual skills (comparator) in the prevention and reduction of workplace violence. Londrina, PR, Brazil, 2021

Source: Graph generated in the RevMan^®^ statistical program^32^; SD = Standard Deviation; IV = Interval of Variance; CI = Confidence Interval

Therefore, no scientific evidence was found for the mean difference of the effect of the IG when compared to the GC (individual skills versus individual skills), according to the forest plot shown in Figure 4.

In Figure 5, certainty of the evidence of the results evaluated by means of the GRADE system was classified as very low for the outcome analyzed (violence prevention/reduction).

**Figure 5:** Synthesis of the assessment corresponding to certainty of the evidence, according to Grading of Recommendations Assessment, Development and Evaluation. Londrina, PR, Brazil, 2021

Certainty assessment							Summary of the results				
Outcome: prevention/reduction of workplace violence in the professionals from the health and support services											
Number of studies	Type of study	Risk of bias	Inconsistency	Indirect evidence	Imprecision	Other considerations	Event rates (%)		Effect		Certainty of the evidence
							Skills development (Intervention)	Skills development (Control)	Relative (95% CI^§^)	Absolute (95% CI^§^)	
2	Randomized clinical trial	Very severe^*^	Very severe^†^	Not severe	Severe^‡^	None	212	197	Not grouped	MD^||^: -0.08 (-0.41 to 0.25)	○○○ Very low

Source: Chart elaborated in and extracted from the GRADEpro^®^ software^34^. ^*^Two levels decreased due to uncertain risk of bias in both studies, in the “intervention deviation” and “general” domains; ^†^Two levels decreased, as I^2^=88%; ^‡^One level decreased due to the difference in the direction of the effect of the confidence intervals of both studies; ^§^CI = Confidence Interval; ^||^MD = Mean Differences

## Discussion

The studies found in this review reveal that most of them (63.7%) were carried out in the United States of America, which may show that there is greater effort to implement guidelines and strategies to combat violence at work in this country. This can be due to the efforts by the Occupational Safety and Health Administration (OSHA) of the United States of America, which was created in 1970 and had its first bulletin published in 2002 warning about zero-tolerance policies for workplace violence, including programs to prevent it. The documents were gradually updated and the last one was published in 2016^8^.

The data of this study, with most of the surveys being carried out in hospital units (54.5%), followed by hospital emergency units (36.4%), specifically with the Nursing team (81.9%), and a majority of females (72.8%), are similar to the existing guidelines and studies, as they assert that workplace violence has predominantly affected the female Nursing team members working in hospitals^8^ ^-^ ^9^ ^,^ ^47^ ^-^ ^48^.

A Brazilian survey carried out in 2017 with municipal professionals confirms that violence predominantly affects women (47.9%) in relation to men (22.0%), including community agents and nurses, among others^10^.

A systematic review with meta-analysis revealed that female nurses are more likely (21%) to being victims of verbal harassment than their male counterparts^49^. In relation to another review, it was verified that there are more chances for women being victims of sexual harassment when compared to men^50^.

Therefore, there is consensus in the studies found on workplace violence in the health sector, which infer that this type of violence, especially cases of verbal and sexual harassment, predominantly affect women and female nurses.

The high prevalence of workplace violence among female health service professionals, as in this systematic review, can be explained by the lack of knowledge about the different forms of this type of violence and to the naturalization of this violence and of the sexist culture, in addition to the preponderance of female professionals in health services.

In this context, measures to combat the prevalence of workplace violence among women are essential, such as awareness campaigns, training sessions, work groups, referral based on established flows in cases of violence, mandatory records of violence, action plans, and implementation of these actions and local and national laws.

Among the existing theories that seek to explain the phenomenon of workplace violence, an interactive and theoretical model shows that it has a multifaceted nature and that it can be understood through the interaction of several interrelated factors such as individual, social, environmental and occupational ones^51^. This theory reveals that the victim’s gender, age, task situation and working without any other colleague (from home) can exert an influence on the occurrence of violence.

Together, other risk factors such as individual issues inherent to the aggressor (e.g., use of illegal substances) and social and contextual factors (e.g., violence in society, negative culture of violence and insecurity at work) can influence the outcome of violence (physical and/or psychological) both for the workers, including stress, diseases and health problems, high costs and suicide, and for the employing institution, including absenteeism and the quality of the care provided to the patients^51^ ^-^ ^52^.

A fact that furthers aggravates the magnitude of the problem of the aforementioned type of violence is its under-reporting^2^ ^,^ ^53^. A study carried out in a North American hospital system with approximately 15,000 employees concluded that 88% of the professionals had not recorded any incidents in the electronic system that is used in the United States of America for notification, where incidents that caused injuries were mainly reported^53^. Thus, under-reporting is even more alarming in cases of harassment and abuse, possibly due to the culture of trivializing violence in the workplace, considered by many to be common in the health sector^51^.

Notification of violence still needs to advance to determine the extent and depth of this problem, including preventive measures and interventions that can support health professionals so that they report violence and are protected against reprisals, both at work and from the aggressor^54^.

The quasi-randomized methodology, that is, studies with two pre- and post- non-equivalent groups (IG and CG), found in most of the studies (63.7%), even without randomization, can answer questions and estimate the success of a health intervention implemented rather than estimating the health effects of this intervention, as in randomized clinical trials^22^. Thus, the low percentage of randomized studies (36.3%) exerted an influence on the estimation of the global effect of the studies presented, which may be improved with a greater number of this methodology.

A research study verified that the clinical outcomes can improve, especially after 12 months of study^22^. Thus, a study^37^ published in 2006 was significant, although only for data on verbal abuse, with a study period of 12 months, 6 months post-intervention (online training). In another study^38^ published in 2009, with 108 months of study, implementation of the policy was able to significantly increase the health professionals’ safety 72 months after the intervention.

For the randomized clinical trials, a study^43^ published in 2017 was also effective and significant in reducing the risks of patient-worker violence and related injuries after the 24-month period since implementation of the intervention, with a 60-month study period. In another randomized clinical trial^42^, computer-based training also reduced incidents of violence and harassment in the workplace, although with no significant difference between the groups. In this study^42^, time corresponded to 6 months, and the intervention took place 2 months after initiating the study. Thus, it is inferred that monitoring time can be related to the significance of the effect of the intervention.

A systematic review conducted with nine randomized and quasi-randomized studies concluded that the diverse scientific evidence is extremely uncertain about the effects of education and training on aggression in the short-term follow-up when compared to no intervention. In the long-term, education was not able to reduce the violence rates in relation to no intervention (low certainty of the evidence)^13^.

The interventions classified in this study were defined according to the International Labor Organization^7^, namely: development of individual skills, environmental and organizational approach, multiple approach (individual, environmental and organizational skills) and governmental actions (implementation of laws/policies/guidelines) at the prevention level and. in this systematic review, the majority (54.5%) corresponded to individual skills, followed by the multiple approach (36.4%).

These data allow inferring that governmental interventions are still incipient and scarce, revealing a weakness in the development of public policies and regulations that prevent workplace violence and, in turn, reduce it worldwide.

Among the randomized clinical trials, two studies^40^ ^,^ ^43^ exerted significant effects on the violence rates. A study^40^ published in 2012 implemented an individual skill approach, including an Internet training session designed to teach strategies through courses with videos demonstrating behaviors, in a one-week period, to nursing assistants, in both groups, IG and CG, but with different intervention times, in order to deal with, prevent or reduce aggressive behaviors by institutionalized aged individuals. In the IG, training was performed immediately after the beginning of the study, while in the CG, it was conducted after 8 weeks, with the study lasting 6 months.

Another research study^43^ conducted during 60 months implemented an action-research (*Plan-Do-Study-Act*). No intervention was implemented in the CG, whereas the IG did undergo interventions. The unit supervisors were instructed to record all the violent events reported in the electronic system up to 24 hours after receiving the notification. Thus, by means of focus groups, the units were presented their violence rates as related to the hospital system of the United States West region. Secondly, an action plan was developed among the professionals, researchers and supervisors, including the implementation and change of this action plan, not only in terms of individual or behavioral factors (focus groups, training sessions), but also of an environmental and organizational nature (records of the workplace violence data, feedback of these data to the units). In the third place, the IG and CG units were monitored 36 months after applying the interventions in order to determine whether measures and improvements were implemented in the units.

This participant research method is widely accepted in health care and consists of a scientific method to test changes in complex environments^55^. A systematic review found that this methodology can be effective, as shown by a study^43^ included in this review, although it needs to be implemented rigorously^55^.

Similarly, two quasi-randomized studies included in this review proved to be effective and significant. A study^37^ found that a three-hour online training session (individual skills development), focusing on violence risk assessment, assertiveness techniques, legal issues and monitoring after an incident, was significant, although only for verbal abuse rates; whereas another study^38^, published in 2009, which implemented governmental intervention (California state law rules), with a period of three years prior to the violence prevention policy and six years of follow-up, inferred that the state law/policy can be an effective method to increase health care workers’ safety. The assault rates among emergency department workers was reduced by 48% in California after enactment of the State Law, when compared to the rates for the New Jersey emergency department, which had only federal guidelines, in this case, from OSHA.

It is therefore understood that the guidelines are essential, but a regulatory norm, with a mandatory character, has greater coercive conditions for the implementation of preventive measures against workplace violence.

The implementation of governmental interventions, including legal aspects or public policies, at the local, municipal, regional, state or national level is an effective strategy with high potential to address workplace violence in the health services in a collective and sustainable way^56^. Such being the case, governmental interventions and efforts must be implemented so that a safe and decent work environment is promoted.

Despite the efforts in different countries or locations, many places do not yet have legislation or public policies that ensure specific strategies to prevent workplace violence against health professionals^56^. In the Brazilian context, an infographic of Nursing photography, performed in 2020, concluded that there are no preventive measures against violence for health professionals in Brazil^57^.

Therefore, it can be asserted that both individual skills and the multiple approach (individual, organizational and environmental) and the implementation of governmental interventions (legislation) can minimize the violence rates and risk, as well as they can improve monitoring of the workplace violence notification systems^58^.

It is noted that the different periods of the interventions implemented in the studies described above can interfere with their effectiveness. In addition to that, effectiveness can also be affected by the follow-up time of the studies, as research has found that the outcomes can improve with increased study follow-up times^22^.

In relation to the risk of bias, no studies were identified with low risk, only with uncertain risk or high risk for a classification of five domains in randomized clinical trials and seven domains in quasi-randomized trials, influencing the final estimate of the effect.

In randomized clinical trials, the concerns, especially in domain 2 (deviations from the intended intervention) and domain 4 (measurement of the outcomes), can be mainly related to blinding of the participants and to incomplete data about the results in the outcome. While in the quasi-randomized trials, the studies had concerns mainly in domain 1 (confounding factors), domain 3 (intended intervention or classification of the intervention) and domain 6 (measurement of the outcomes).

Thus, both methodologies presented problems both in the intended intervention and in measurement of the outcomes. It is emphasized that workplace violence is complex and often requires the professional’s report; therefore, it can be difficult to adopt masking of the participants.

The meta-analysis allowed inferring that there was no improvement in the outcome of the intervention (individual skills) *versus* the comparator (individual skills) for the random effect model, with individual skills including training sessions, programs and courses. Such being the case, there was no scientific evidence that the individual skills intervention may prevent or reduce workplace violence (95% CI: -0.41 - 0.25, p=0.64, I^2^=88%), when compared to the control condition. 

The lack of scientific evidence and the high heterogeneity between the meta-analysis studies can be explained by the difference in the direction of the effect of the confidence intervals of the studies included. Another explanation can be due to the fact that the same strategy was implemented in the intervention and control groups (individual skills). Consequently, it was not possible to infer which intervention was more effective for the outcome evaluated.

A systematic review without meta-analysis carried out with 15 studies revealed that most of them exerted a positive effect on preparing the team to deal with violent situations or on reducing the number of violent incidents, although the evidence is still scarce^59^.

In this same aspect, a systematic review with meta-analysis carried out with nine studies (clinical trials and quasi-randomized studies) found that the evidence from the studies is still extremely uncertain about the effects of education and training against aggression when compared to no intervention^13^.

Similarly, certainty of the evidence of the outcome evaluated in this review by means of the GRADE system was classified as very low, as the risk of bias of the studies found (uncertain) and the inconsistency (I^2^=88%) or high statistical heterogeneity were the main factors that determined certainty of the evidence of the studies evaluated as very low.

The lack of recent studies stands out among the limitations of this systematic review, as only four studies were published in the last three years, 2017-2019, revealing the need to carry out new randomized clinical trials and quasi-randomized studies. In addition, non-standardization of the analysis of the age of the samples of the studies, as some of them evaluated the total mean or the mean per group, while others assessed the predominant age group; and omission of the participants’ gender in three studies also represented a limitation.

The reduced number of randomized clinical trials was also a limitation, in addition to the fact that only two of these studies were homogeneous in terms of PICO, which may have hindered generalizing the findings and replicating the evidence for the practice. The quasi-randomized studies found may have reduced the chances of showing effectiveness of the implemented interventions due to interrupted time series and poorly controlled confounding variables.

Thus, this study contributed in an attempt to deepen knowledge and respond to the study object, aiming to contribute to filling the gap in the literature on this theme. Systematic reviews with meta-analysis are useful to determine the knowledge synthesis and the effectiveness of the interventions. The results found provide subsidies for workers, managers and public policy makers to implement interventions for the prevention and reduction of workplace violence.

That said, this study collaborated and advanced to deepen knowledge about the effectiveness of preventive actions and that reduce acts of violence against health and support professionals, contributing to filling the gap in the literature on this theme. It also provides subsidies for workers, managers and public policy makers to implement interventions for the prevention and reduction of the workplace violence suffered by health and support professionals.

## Conclusion

This review did not reveal high scientific evidence for the outcomes of prevention and reduction of workplace violence in relation to the interventions (individual skills, multiple approach and governmental actions) implemented, mainly due to the high and uncertain risk of bias in the studies, in addition to the high statistical heterogeneity.

Due to the impossibility of a precise scientific judgment in this meta-analysis, it is recommended to conduct more randomized clinical trials with standardization of the interventions, low risk of bias and low consistency, so that the most effective interventions can be replicated in the practice and provide a safe and decent work environment for health and support professional.
